# Analysis of Proteasomal Proteolysis during the *In Vitro* Metacyclogenesis of *Trypanosoma cruzi*

**DOI:** 10.1371/journal.pone.0021027

**Published:** 2011-06-17

**Authors:** Josiane Cardoso, Carla De Paula Lima, Tiago Leal, Daniela F. Gradia, Stênio P. Fragoso, Samuel Goldenberg, Renata Guerra De Sá, Marco A. Krieger

**Affiliations:** 1 Instituto Carlos Chagas/FIOCRUZ, Curitiba, Parana, Brazil; 2 Universidade Federal de Ouro Preto/UFOP, Ouro Preto, Minas Gerais, Brazil; Federal University of São Paulo, Brazil

## Abstract

Proteasomes are large protein complexes, whose main function is to degrade unnecessary or damaged proteins. The inhibition of proteasome activity in *Trypanosoma cruzi* blocks parasite replication and cellular differentiation. We demonstrate that proteasome-dependent proteolysis occurs during the cellular differentiation of *T. cruzi* from replicative non-infectious epimastigotes to non-replicative and infectious trypomastigotes (metacyclogenesis). No peaks of ubiquitin-mediated degradation were observed and the profile of ubiquitinated conjugates was similar at all stages of differentiation. However, an analysis of carbonylated proteins showed significant variation in oxidized protein levels at the various stages of differentiation and the proteasome inhibition also increased oxidized protein levels. Our data suggest that different proteasome complexes coexist during metacyclogenesis. The 20S proteasome may be free or linked to regulatory particles (PA700, PA26 and PA200), at specific cell sites and the coordinated action of these complexes would make it possible for proteolysis of ubiquitin-tagged proteins and oxidized proteins, to coexist in the cell.

## Introduction

Proteasomes are the principal non-lysosomal degradation machinery present in all types of eukaryotic cells [1], [2]. The proteasome system regulates many cellular functions including cell cycle progression, cell differentiation, stage-specific gene transcription [3], antigen processing [4], the regulation of membrane-anchored and secretory pathway-compartmentalized proteins [5], as well as protein quality control [6].

The 20S proteasome is the catalytic core of this degradation machinery. It is present in a latent form in the cell, but it is activated by various types of regulatory complexes [7]. Proteasome subtypes with different proteolytic properties are formed by the attachment of these regulatory complexes to one or both endplates of the barrel-shaped 20S core particles [8]. This proteasome is a threonine protease with its active sites located within the particle. In eukaryotes, two of these sites are chymotrypsin-like, two are trypsin-like, and two are caspase-like [9]. For threonine protease activity, the hydroxyl group of the terminal threonine residue of the beta subunit acts as the catalytic nucleophile, attacking and degrading peptide bonds into small peptides of 3 to 20 residues that are further hydrolyzed by other peptidases [9]. The physiological function of the 20S proteasome has not been clearly defined, but it is well documented that it degrades oxidized proteins independently of ATP and ubiquitin [10]–[14].

The 20S proteasome binds to the 19S regulatory complex in an ATP-dependent manner, to form the 26S proteasome. The 19S complex consists of about 20 subunits, including six ATPase subunits. The 26S form of the proteasome is responsible for degrading most of the short-lived cellular proteins, through ubiquitin-dependent proteolysis [15]. The 11S regulator, known as the PA28 or PA26 complex in mammals and *Trypanosoma brucei,* respectively, are heptamers that stimulate 20S proteasome peptidase activity in an ATP-independent manner [16]. PA26 complexes stimulate the degradation of small peptides, but not ubiquitinated proteins [16]. The PA28 complex is induced by γ-interferon and is required for efficient antigen processing [17].

The relative contributions of the different proteasome complexes vary between cell types and, despite intensive research, the functional significance of the different complexes found in eukaryotic cells remains unclear [15].

*Trypanosoma cruzi*, the causative agent of Chagas' disease, undergoes profound morphological changes during its development in vertebrate and invertebrate hosts. The process by which non-infectious, replicative epimastigotes are transformed into infectious, non-proliferative metacyclic trypomastigotes is called metacyclogenesis. It remains unclear how the differentiation process is triggered, but it has been demonstrated that metacyclogenesis can be induced by nutritional stress [18]–[20]. The parasite response to environmental changes, such as nutrient availability, is correlated with changes in gene expression. In trypanosomatids, the regulation of gene expression is mostly post-transcriptional, but little is known about the specific mechanisms involved in adaptive protein turnover.

We have shown that the inhibition of proteasome activity blocks *T*. *cruzi* growth and metacyclogenesis [21]. Furthermore, intracellular amastigote-to-trypomastigote transformation is prevented by specific proteasome inhibitors [22], [24]. Thus, proteasome activity is required for cell remodeling and probably plays an important role in parasite development. Indeed, several studies have shown that cell differentiation is dependent on intracellular proteolysis [22], [24]–[26].

In this study, we assessed the contribution of proteasomes to proteolytic degradation during *T. cruzi* cell differentiation. We investigated the enzymatic activity of the proteasomes, and the intracellular localization of the proteins present in these complexes (the catalytic subunit, alpha 7, of the 20S proteasome; the RPN10 protein from regulatory subunit 19S and PA26 protein from the proteasome activator 26). We also evaluated the profile of ubiquitinated conjugates and oxidized proteins during metacyclogenesis. Our results suggest that, in *T. cruzi*, the ubiquitin-independent mechanism of protein degradation mediated by proteasomes operate during metacyclogenesis.

This work provides a framework for understanding the mechanisms underlying the various degradation routes mediated by proteasomes. An evaluation of proteasomal degradation during cell differentiation provides a global view of the presence and dynamics of the different complexes involved in protein degradation in the cell.

## Results

### Western blot analysis of alpha 7, RPN10 and PA26 during metacyclogenesis *in vitro*

**Figure 1:**
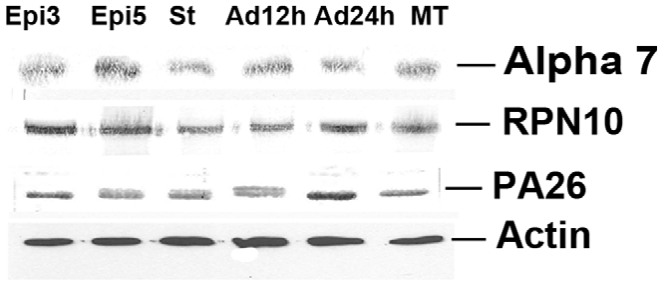
Western blot analysis of alpha 7, RPN10 and PA26 during metacyclogenesis. Protein extracts (20 µg of protein) from three-day-old cultured epimastigotes (Epi 3d), five-day-old cultured epimastigotes (Epi 5d), five-day-old cultured epimastigotes under nutritional stress (Epi St), adhered epimastigotes after 12 h of differentiation (Adh 12 h), adhered epimastigotes after 24 h of differentiation (Adh 24 h) and metacyclic trypomastigotes (MT) were analyzed by Western blotting with antisera directed against alpha 7, RPN10 and PA26. The experiment was carried out three times, with essentially the same results in each case.

Specific antibodies directed against the catalytic subunit alpha 7 of the 20S proteasome, the regulatory subunit 10 (RPN10) of 19 S and the proteasome activator 26 (PA26) were used in western blot assays with protein extracts collected during metacyclogenesis. The proteins Alpha 7, RPN10 and PA26 were detected in similar levels throughout the cellular differentiation process (Figure 1).

### Immunolocalization of alpha 7, RPN10 and PA26

**Figure 2:**
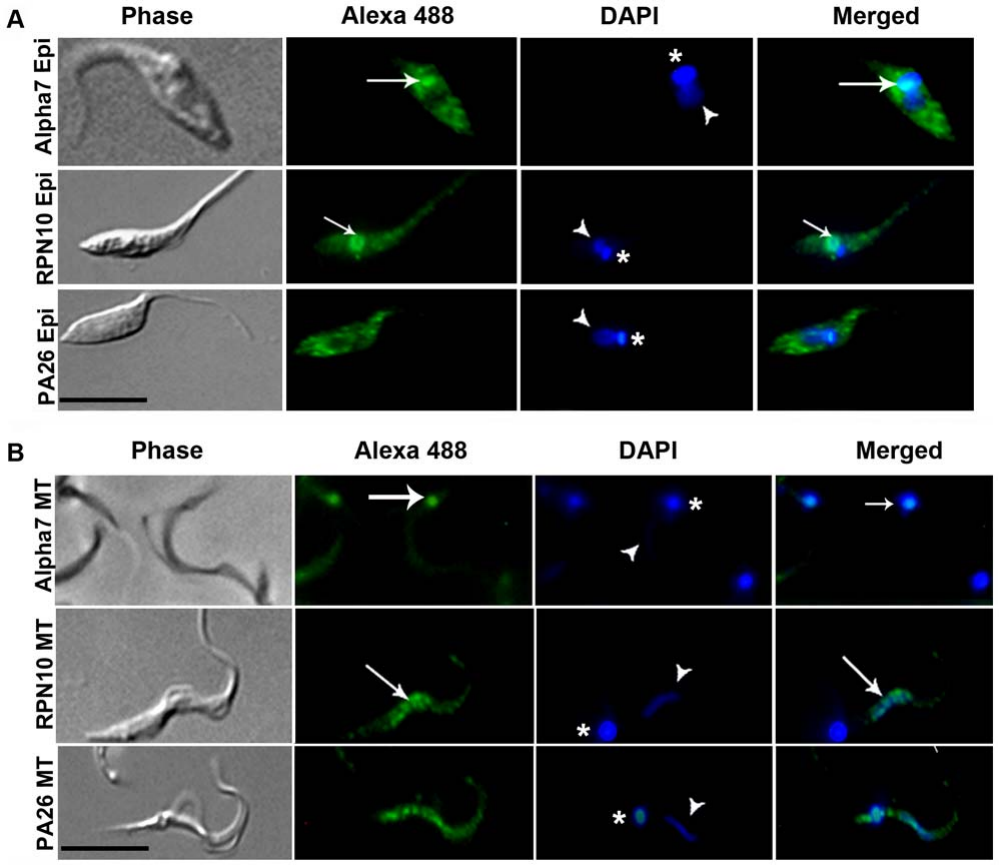
Intracellular localization of proteasomal proteins. Parasites were labeled with antibodies specific to the catalytic subunit 7 of 20S proteasome (alpha 7) regulatory subunit 10 of 19S (RPN10) and activator proteasome 26 (PA26) in (A) three-day-old cultured epimastigotes (Epi) and (B) metacyclic trypomastigotes (MT). DAPI were used to stain nuclei (arrow head) and kinetoplast (asterisk). Merged images suggesting co-localization of alpha 7 (20S proteasome) with kinetoplast (arrow) of epimastigotes and mainly metacyclic trypomastigotes and co-localization of RPN10 (19S complex) with nuclei (arrow) of epimastigotes and metacyclic trypomastigotes and co-localization of RPN10 (19S complex). Bars = 10 µm.

To evaluate the intracellular distribution of proteasomes in parasites we used specific antibodies directed against the catalytic subunit alpha 7 of the 20S proteasome, regulatory subunit 10 (RPN10) of 19 S and proteasome activator 26 (PA26) in epimastigotes (Figure 2A) and metacyclic trypomastigotes (Figure 2B).

The immunolocalization of catalytic subunit alpha 7 revealed a distribution in the cytoplasm, and a strong reaction with kinetoplast of epimastigotes (Figure 2A). In parasites metacyclic trypomastigotes a predominantly kinetoplast localization was observed (Figure 2B).

When epimastigotes were examined by microscopy after incubation with antibodies directed against the regulatory subunit RPN10, a granular distribution was observed, as well as a strong and specific reaction with nuclei (Figure 2A - arrow). The same results were observed in metacyclic trypomastigotes (Figure 2B - arrow).

PA26 appears in a granular distribution in the cytoplasm and kinetoplast, with points of accumulation throughout the parasite body in epimastigotes. Although a similar distribution was observed in metacyclic trypomastigotes, we also detected a nuclear labeling pattern in these cells.

### Proteasomal activity during *T. cruzi* metacyclogenesis *in vitro*

To show the presence of proteasome activity during differentiation, peptidase activities during in vitro metacyclogenesis were determined by the hydrolysis of specific fluorogenic substrates. In three-day-old cultured epimastigotes, five-day-old cultured epimastigotes, epimastigotes under nutritional stress and adhered epimastigotes after 12 h and 24 h of differentiation, the trypsin-like and chymotrypsin-like activities were increased about twice when compared to metacyclic trypomastigotes (Table 1).

**Table 1:** 20S proteolytic activity during *T.cruzi in vitro* metacyclogenesis.

Relative Fluorescence Units						
Groups	three-day-old epimastigotes	five-day-old epimastigotes	epimastigotes under nutritional stress	adhered epimastigotes after 12 h	adhered epimastigotes after 24 h	metacyclic trypomastigotes
Peptidase activities						
Chymotrypsin-like	11.3±2.86	12.0±1.6	11.9±0.235	9.75±1.02	10.1±1.05	6.2±0.315
Chymotrypsin-like + MG132	0.38±0.33	0.43±0.4	0.61±0.67	0.32±0.25	0.63±0.47	0.27±0.30
trypsin-like	9.85±1.19	11.7±1.51	13.1±1.024	11.4±1.515	11.8±1.125	4.92±0.525
trypsin-like + MG132	4.25±0.49	4±0.98	4.55±1.48	4.85±1.9	4.1±0.28	2.7±0.56
caspase-like	0.26±0.01	0.2±0.17	0.42± .051	0.43±0.168	0.54±0.098	0.07±0.042
caspase-like + MG132	0.08±0.02	0.09±0.007	0.14±0.08	0.13±0.08	0.105±0.13	0.05±0.01

Peptidase activities were assayed using 100 µg of proteins of crude extratct from each of the six stages of cell differentiation. Chymotrypsin-like, trypsin-like, and caspase-like activities in presence or absence of MG132, were determined by fluorimetric quantification of the substrates Z-Gly-Gly-Arg-AMC, Suc-Leu-Leu-Val-Tyr-AMC and N-Cbz-Leu-Leu-Glu-β-NA, at 380 nm excitation/440 nm emissions, respectively. The results are presented in Relative Fluorescence Unit (RFU). Values shown are mean ± standard deviation from three independent experiments.

**Figure 3:**
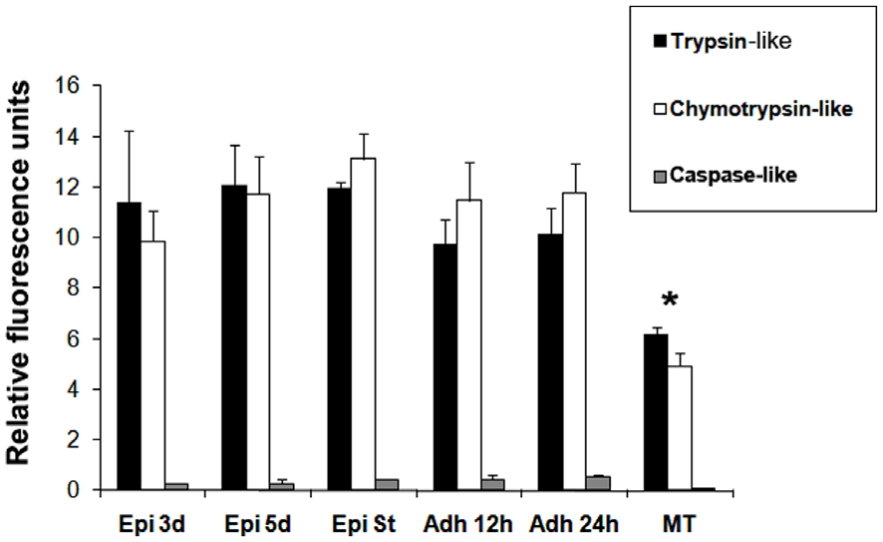
Proteasome proteolytic activity during *T.cruzi* metacyclogenesis *in vitro.* Peptidase activities were determined by fluorimetric quantification of the hydrolysis of specific fluorogenic substrates: trypsin-like (black bars), chymotrypsin-like (white bars) and caspase-like (gray bars) proteases. Data is shown as the activity in each parasite group analyzed in three independent experiments: three-day-old cultured epimastigotes (Epi 3d); five-day-old cultured epimastigotes (Epi 5d), five-day-old cultured epimastigotes under nutritional stress (Epi St), adhered epimastigotes after 12 h of differentiation (Adh 12 h), adhered epimastigotes after 24 h of differentiation (Adh 24 h), and metacyclic trypomastigotes (MT). Data were analyzed by multifactor ANOVA followed by Fisher's test. Statistically significant differences between groups are indicated with an asterisk (*). Differences were considered significant if the p value obtained was less than 0.05.

The fluorescence measured in the presence of the proteasome inhibitor MG132 was used as a control; in these conditions, we observed 95% inhibition of the chymotrypsin-like activity and 30% inhibition of the trypsin-like and caspase-like activities (Figure S1). There is proteasome activity in all differentiation stages during metacyclogenesis, trypsin-like and chymotrypsin-like activities were high, whereas caspase-like activity was low (Figure 3).

Proteolytic activity was higher in the epimastigote group when compared to metacyclic trypomastigotes (Figure 3).

### Ubiquitination is constitutive during metacyclogenesis *in vitro*

**Figure 4:**
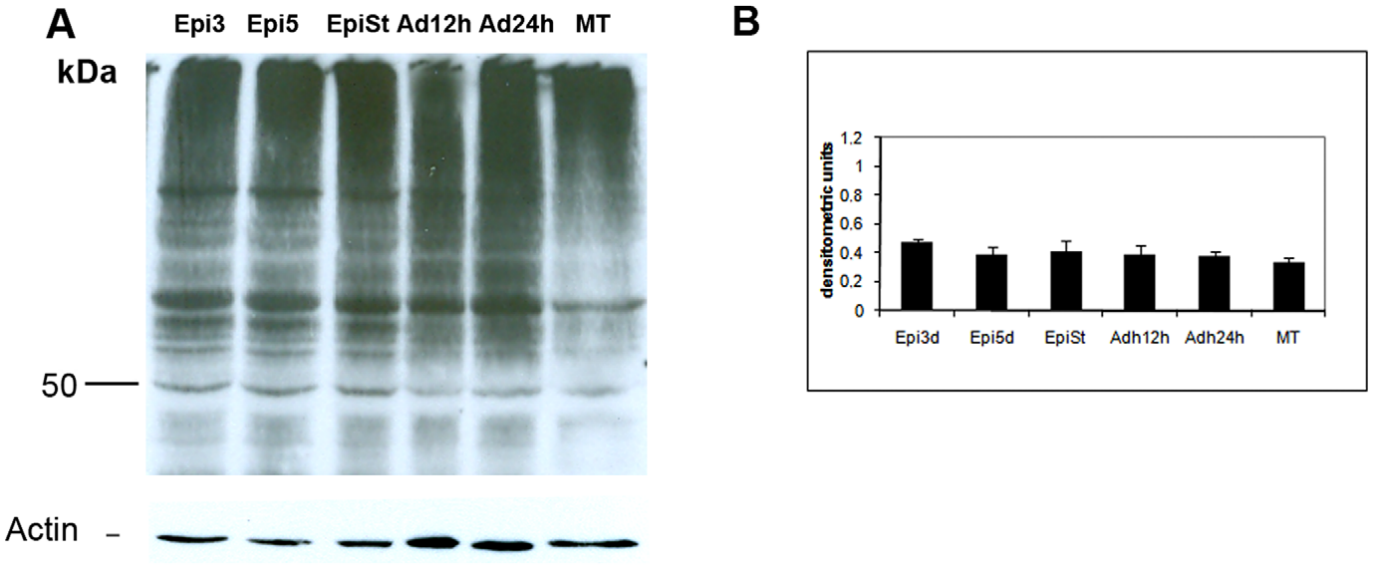
Ubiquitination during metacyclogenesis. We analyzed protein extracts (20 µg of protein) from three-day-old cultured epimastigotes (Epi 3d), five-day-old cultured epimastigotes (Epi 5d), five-day-old cultured epimastigotes under nutritional stress (Epi St), adhered epimastigotes after 12 h of differentiation (Adh 12 h), adhered epimastigotes after 24 h of differentiation (Adh 24 h), and metacyclic trypomastigotes (MT) by western blotting with a mouse antiserum directed against the ubiquitin of *T. cruzi*. Data were normalized with respect to actin, as described in the Methods. Protein molecular weight is shown on the left. The experiment was carried out three times, with essentially the same results in each case.

Since proteasomal proteolysis varies during metacyclogenesis, we evaluated the ubiquitin profile during this cellular differentiation process. Ubiquitinated conjugates profile remained similar throughout metacyclogenesis (Figure 4). No evidence of peaks of ubiquitination was found at any specific stage and the profile remained constant. Densitometry data were normalized with respect to actin content and no significant differences were found between the differentiation stages (p>0.05).

### Protein carbonyl group content varies during metacyclogenesis *in vitro*

Once ubiquitin profile did not change during differentiation and proteasome proteolytic activity varied, we asked if ubiquitin independent degradation, such as degradation of oxidized proteins, could be altered during this process. The oxidized proteins are natural substrates of ATP/ubiquitin-independent degradation by 20S proteasome. The oxidized protein levels during metacyclogenesis were estimated by determining the levels of protein carbonyl groups.

**Figure 5:**
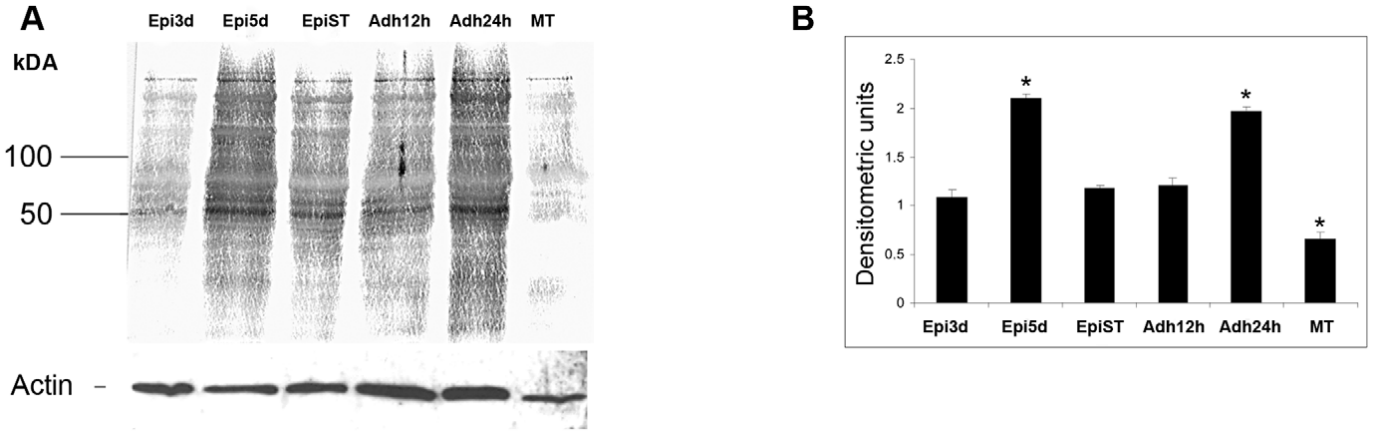
Detection of protein carbonyl content. Protein carbonyl groups were labeled with 2,4-dinitrophenylhydrazine and the resulting samples (20 µg of protein) were subjected to western blotting with an antibody against dinitrophenyl: three-day-old cultured epimastigotes (Epi 3d), five-day-old cultured epimastigotes (Epi 5d), five-day-old culturd epimastigotes under nutritional stress (Epi St), adhered epimastigotes after 12 h of differentiation (Adh 12 h) adhered epimastigotes after 24 h of differentiation (Adh 24 h) and metacyclic trypomastigotes (MT). Significant differences between groups are indicated with an asterisk (*). Protein molecular weight is shown on the left. Normalization was performed as described in the Methods. The experiment was carried out three times, with essentially the same results in each case. Results are expressed in densitometric units and are the means of three independent experiments ± SD.

Considerable differences were found between the various parasite forms studied (Figure 5). Protein carbonyl content was significantly higher in five-day-old epimastigotes and adhered epimastigotes after 24 h of differentiation (lanes 2 and 5) than in three-day-old epimastigotes, epimastigotes under nutritional stress, adhered epimastigotes after 12 h of differentiation (lanes 1, 3 and 4) and metacyclic trypomastigotes (lanes 6).

Densitometry data were normalized with respect to actin content and significant differences were found between differentiation stages (p>0.05).

### Protein carbonyl content increases with proteasome inhibition

**Figure 6:**
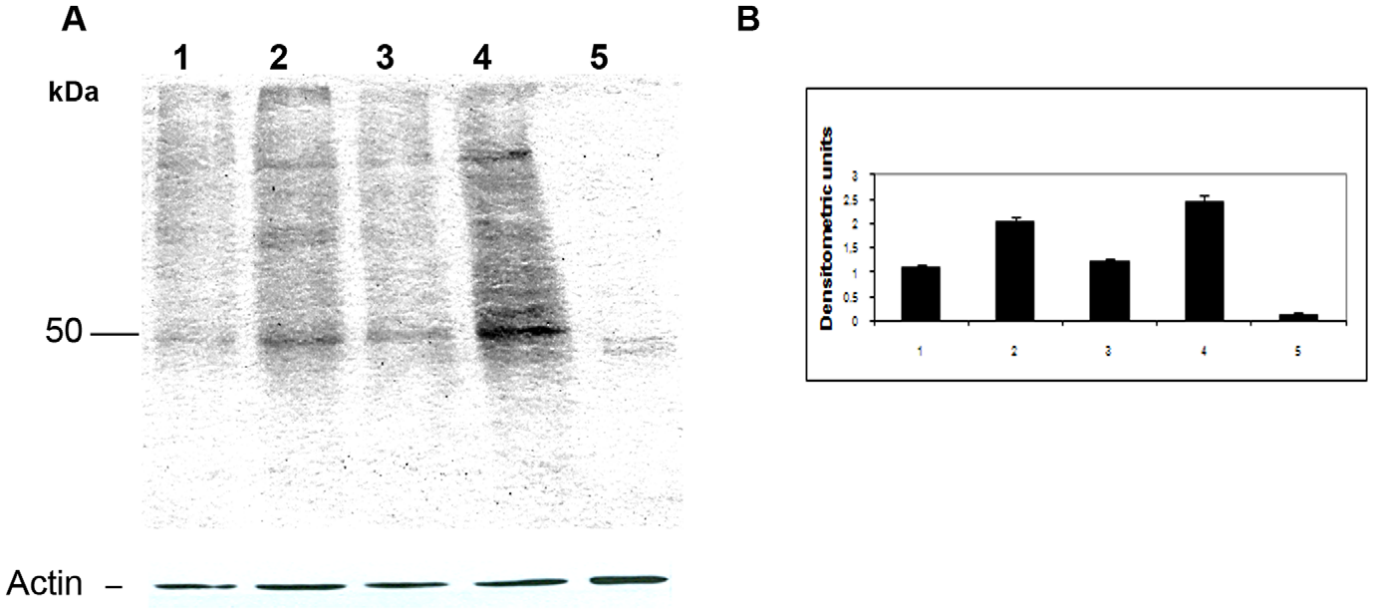
Detection of protein carbonyl groups after proteasome inhibition. Protein carbonyl groups were labeled with 2,4-dinitrophenylhydrazine and the resulting samples (20 µg of protein) were subjected to western blotting with an antibody against dinitrophenyl: control epimastigotes (without drug) after 48 h in LIT medium (1); epimastigotes treated with 5 µM lactacystin for 48 h in LIT medium (2); control epimastigotes (without drug) after 72 h in LIT medium (3); epimastigotes treated with 5 µM lactacystin for 72 h in LIT medium (4); epimastigotes treated with 5 µM lactacystin for 72 h in LIT medium without 2,4-dinitrophenylhydrazine labeling (reaction control) (5). Normalization was carried out as described in the Methods. The experiment was carried out three times, with essentially the same results in each case. Results are expressed in densitometric units and are the means of three independent experiments ± SD.

To demonstrate that changes in oxidized proteins profile could be affected by proteasome, this activity was blocked with lactacystin a specific proteasome inhibitor. The protein carbonyl content in parasites treated with lactacystin for 48 or 72 h was higher than in control parasites (Figure 6). This experiment showed that protein carbonyl content is also modulated by proteasome.

The control sample did not react with the antibody indicating that there was no non-specific reaction. Densitometry data were normalized with respect to actin content, and significant differences were observed between treated and control samples (p>0.05).

## Discussion

Proteasomes are responsible for most of the non-lysosomal protein degradation occurring in eukaryotic cells [27], [28]. Several studies have demonstrated that proteasome activity is required for cell remodeling and the development of parasitic protozoa [22], [24]. We have previously shown that the inhibition of proteasome activity blocks the proliferation of epimastigotes and the differentiation of epimastigotes into metacyclic trypomastigote forms of *T. cruzi* [21].

The *T. cruzi* genome includes the genes encoding all 14 subunits of proteasome 20S and all the regulatory subunits of 19S (PA700) PA26 and PA200, but no molecular studies have been carried out during cell differentiation to evaluate activation of the various proteasome complexes and their contribution to proteolytic degradation.

We measured the three proteolytic activities of the proteasome (trypsin-like, chymotrypsin-like and caspase-like) with fluorogenic exogenous substrates. We performed the assay with crude cell extracts that had previously been treated with several protease inhibitors to ensure that all major serine, cysteine and calcium-dependent proteases were inhibited. We can therefore conclude that the proteolylic activity measured corresponds mainly to the proteasome, because it has already been shown that neither cysteine and serine protease inhibitors nor EDTA affects proteasome activity [22]. Our data show that proteasome-dependent proteolysis occurs during metacyclogenesis. The proteasome is active in all phases of differentiation, with trypsin-like and chymotrypsin-like activities higher than caspase-like activities in all parasite groups (Figure 1). These results confirm the observations reported in a recent study of *T. cruzi* [23]. The proteasome inhibitor MG132, used as a control, efficiently inhibited all peptidase activities [29]. The observed difference in peptidase activities is consistent with heterogeneity in protozoan parasite proteasomes [30].

We observed that there were no significant changes in the ubiquitinated conjugates profiles at all stages of differentiation. This suggests that ubiquitination might be a constitutive mechanism and that the conditions during in vitro metacyclogenesis did not alter this system. It is well known that protein ubiquitination is not only a degradation pathway, but also a signaling pathway which does not involve protein degradation [31].

In contrast, carbonylated protein determinations revealed significant variation in the levels of oxidized proteins among the *T. cruzi* differentiation stages. Epimastigotes of 5-day-old are at late logarithmic growth phase preparing for the differentiation process while adhered epimastigotes of 24 h, which are in the final stages of differentiation, are being released as metacyclic trypomastigotes into the medium. We speculate that, due to the physiological importance of these two stages in the differentiation process, the higher level of oxidized proteins observed could be related to the need for selective proteins degradation. It is known that the proteasomal system is the major proteolytic pathway responsible for the removal of oxidized proteins in an ubiquitin-independent manner [31]. Indeed, we observed differences in proteasomal proteolytic activity between stages (Figure 3), which also correlate to the metabolic changes described in the literature [34]. Furthermore, proteasome inhibition in epimastigotes caused an increase of oxidized protein content (Figure 6). It should be noted that proteasome inhibition during metacyclogenesis results in complete inhibition of the differentiation process [21]. Taken together, these results suggest that proteasomes are involved in oxidized protein degradation during *T. cruzi* metacyclogenesis.

A comparison between epimastigotes and metacyclic trypomastigotes at proteomical levels indicates an increase in the production of proteins involved in antioxidant defenses [32]. Several proteins, with distinct regulation status, were identified as responsible for maintaining the cell redox status during metacyclogenesis [33]. Thus, physiological stress conditions associated with *T. cruzi* differentiation [34] may modify the redox status of the parasite by activating and/or inactivating metabolic pathways. In this context, proteasomes could be regulating cellular homeostasis by degradation of oxidized proteins during metacyclogenesis in vitro.

Possibly, several proteasome conformations may work together in protein degradation during *T. cruzi* metacyclogenesis. The catalytic subunit alpha 7 of the 20S proteasome was observed more intensely in kinetoplasts of epimastigotes and metacyclic trypomastigotes. Recently the presence of active proteosomes in the kinetoplast of T.cruzi epimastigotes was shown [23]. The analysis revealed the presence of the catalytic subunit alpha 5 of the 20S proteasome in the nucleus, cytoplasm and also in the kinetoplast of epimastigotes. The electron microscopy assay showed a more intense presence in the kinetoplast of metacyclic trypomastigotes [23], as observed in our results.

During *T. cruzi* metacyclogenesis, the kinetoplast is one of the most extensively remodeled structures, changing from a basket-like to a round shape. Kinetoplast remodeling probably involves the expression of new sets of proteins and the degradation of old ones. Oxidized proteins are constantly generated and degraded in the mitochondria [35]. Our results indicate that proteasome proteolysis could be involved in these processes.

The 20S proteasome has been widely reported to degrade oxidized and chemically unfolded proteins in an ATP/ubiquitin-independent manner, whereas the 26S proteasome displays only minimal selective degradation of oxidized proteins [10], [36]–[39].

Our findings on the cellular localization of RPN10 showed distribution in the cytoplasm, along the flagellum and mainly in the nucleus of epimastigotes and metacyclic trypomastigotes. The regulatory subunit 10 is part of the regulatory complex PA700 (19S). This binds to the 20S to form the 26S proteasome, which degrades preferentially proteins targeted by ubiquitin, known as the ubiquitin-proteasome system (UPS). Several studies have shown that various components of the UPS reside in the cell nucleus. The proteasome 26S is involved in DNA repair, replication and transcription [40]–[44].

The data of intracellular localization to PA26 show a granular distribution with points of accumulation throughout the body of the parasite. PA26 complex activates the 20S proteasome in an ATP-independent manner and stimulates the degradation of small peptides, but not ubiquitinated proteins [16].

Proteasomes are active throughout the metacyclogenesis and proteins alpha 7, RPN10 and PA26 are present, without great variations in their levels being noted throughout this process. However, there are points of accumulation throughout the body of the parasite or accumulation in organelles, such as kinetoplast and nucleus, in epimastigotes and metacyclic trypomastigotes. This probably reflects the need for an increase in the proteolysis of specific substrates in these intracellular locations. These data suggest that proteasomes coexist in the cell during metacyclogenesis, being activated at specific sites, as needed.

Finally, our findings strongly suggest that the coordinated series of biochemical adaptations occurring during *T. cruzi* metacyclogenesis may also be regulated by proteasome activity with different proteasome complexes. The 20S proteasome may be free or linked to regulatory particles (PA700, PA26 and PA200), and the coordinated action of these complexes would make it possible for proteolysis, ubiquitin-tagged proteins and oxidized proteins to coexist in the cell. Our data highlight the importance of ubiquitin-independent proteasomal degradation during metacyclogenesis. Additional studies aiming to identify the substrates degraded via ubiquitin-dependent or ubiquitin-independent mechanisms are currently underway in our laboratory.

## Materials and Methods

### Parasite

*Trypanosoma cruzi* strain Dm28c [45] was used. Cultured epimastigote forms were maintained at 28°C in liver infusion-tryptose (LIT) medium [46] supplemented with 10% fetal bovine serum, with weekly passages.

### Epimastigote-to-trypomastigote differentiation

For *in vitro* metacyclogenesis, 5-day-old cultured epimastigotes (5-6×10^7^ cells/ml) were harvested by centrifugation at 7,000 g for 5 min at 10°C. The cells were incubated for 2 h at 28°C in TAU medium (190 mM NaCl, 17 mM KCl, 2 mM MgCl_2_, 2 mM CaCl_2_, 8 mM phosphate buffer pH 6.0), at a density of 5×10^8^ cells/ml (epimastigotes under nutritional stress), and then diluted 1/100 in TAU3AAG medium (TAU supplemented with 10 mM L-proline, 50 mM L-sodium glutamate, 2 mM L-sodium aspartate and 10 mM D-glucose) in TPP tissue culture flasks, in such a way that the added culture medium was no deeper than 1 cm. The cultures were then incubated in TAU3AAG for various time periods. Differentiating epimastigotes (cells attached to the substrate after 12 and 24 h) were obtained by discarding the supernatant and vigorously shaking the flasks with 10 ml TAU3AAG at room temperature to detach the adhered epimastigotes from the flask walls. Metacyclic trypomastigotes were obtained from the TAU3AAG culture supernatant after 72 h of incubation and purified by DEAE-51 chromatography [47]. The parasites were immediately used for the preparation of cell extracts.

### Cloning and production of the recombinant proteins RPN10, alpha 7, PA26 and ubiquitin from *T. cruzi*

The genes encoding the proteasome regulatory non-ATPase subunit RPN10 of *T. cruzi* (Tc00.1047053509611.160; 46 kDa), the catalytic subunit alpha 7 (Tc00.1047053507775.50; 26 kDa), proteasome activator 26 PA26 (Tc00.1047053511465.10; 26 KDa) and ubiquitin (Tc00.1047053506655.20, 19-246 nucleotides 8 kDa) were obtained by searching the *T. cruzi* genome database at TIGR (The Institute for Genomic Research, http://www.tigr.org).

The coding regions of RPN10 were amplified with the primers RPN10F (5′GGGGGATCCTTTCTGTGCCTGGACTCCACGGAGTTTAG- 3′) and RPN10R (5′-GGGGTCGACTTATTTTTTGTTCCTCGGTTGCTTCTCAC-3), and alpha 7 with alpha7F (5′ AACGGATCCTGACCAGTCAACGGACATCTTCTC 3′) and alpha 7R (5′ CAAGTCGACTTGTTGCACACCCATGCCACCT 3′).The ubiquitin gene was amplified with the primers UbF (5′GGGACAAGTTTGTACAAAAAAGCAGGCTTCATGCAGATCTTTGTGAAGACACTG-3′) and UbR (5′ GGGGACCACTTTGTACAAGAAAGCTGGGTCCGCCGCCGCGCAGGCGCAG -′), and PA26 with PA26F (5′GGGGACAAGTTTGTACAAAAAAGCAGGCTTCATGCCGCCAAAACGCCTCGT 3′) and PA26R (5′GGGGACCACTTTGTACAAGAAAGCTGGGTCTTAACTCACCATACGACCTCCCCCGGA 3′).

PCR was performed by mixing 100 ng of total DNA from *T. cruzi* Dm28c, 10 pmol of each primer, 200 µM dNTPs, 1.5 mM MgCl_2_, *Taq* DNA polymerase buffer and 2.5 units of *Taq* DNA polymerase (Invitrogen). The reaction mixture was heated for 4 min at 94°C and then subjected to 35 cycles of denaturation at 92°C for 30 s, annealing at 55°C for 30 s (for the proteasome regulatory non ATPase and catalytic subunit gene) or at 60°C (for the ubiquitin gene) and extension at 72°C for 1 min. The PCR-amplified DNA fragments encoding the proteasome regulatory non-ATPase subunit 10 and the catalytic subunit alpha 7 were inserted into pQE30 (Qiagen), and *Escherichia coli* M15 was transformed with the resulting product. The PCR-amplified DNA fragments encoding ubiquitin and PA26 were inserted into the Gateway platform via the pDONR 221 (Invitrogen) entry vector, and the entry clones were inserted into the pDEST 17 (Invitrogen) destination vector for expression in *E. coli* BL21pLysE (as recommended by the manufacturer). His-tagged recombinant proteins were produced in both *E. coli* strains after the addition of 2 mM IPTG and induction for 4 h at 37°C. The recombinant proteins were purified in denaturing conditions, on Ni–NTA resin (Qiagen), according to the manufacturer's instructions.

### Production of polyclonal antiserum and western blot assay to RPN10, alpha 7, PA26 and ubiquitin from *T. cruzi*

Mice were used to produce polyclonal antisera against the recombinant His6-tagged RPN10, alpha 7, PA26 and ubiquitin proteins. They were immunized by i.p. injection of 20 µg of the appropriate antigen in Freund's complete adjuvant (FCA; Sigma) for the first inoculation, and with 20 µg of the recombinant protein in Freund's incomplete adjuvant (Sigma) for three booster injections, administered at two-week intervals. Antisera were obtained five days after the last booster injection.

Animal experiments were approved by the institutional review board of the Oswaldo Cruz Foundation (CEUA/FIOCRUZ, Protocol number P-0434/07).

For western blot analysis, we subjected about 20 µg of total cellular extract from each of the six stages of cell differentiation to SDS-PAGE and then blotted the gel onto nitrocellulose membranes (Hybond-C, Amersham) according to standard procedures [50]. The membranes were blocked with 5% fat-free milk in TBS buffer (150 mM NaCl, 10 mM Tris-HCl, pH 8.0) supplemented with 0.1% Tween-20 and incubated in the presence of an antiserum against regulatory non-ATPase subunit 10, catalytic subunit alpha 7 or PA26 of *T. cruzi*.

The membranes were washed thoroughly in TBS and incubated with alkaline phosphatase-conjugated goat anti-mouse IgG (Sigma) diluted 1/10,000 and the color reaction was developed with BCIP/NBT (Promega).

### Immunofluorescence assay

Parasite epimastigotes and metacyclic trypomastigotes were washed and resuspended in PBS at a density of 10^7^ cells/ml. The cells were adhered to poly-L-lysine-coated slides for 20 min at room temperature, fixed with 4% paraformaldehyde for 10 min, washed in PBS and then treated with 50 mM NH_4_Cl for another 10 min. Fixed cells were permeabilized with 0.1% Triton X-100 in PBS for 2 min and then blocked by overnight incubation with 1.5% bovine serum albumin (BSA) in PBS. The cells were then incubated for 1 h with polyclonal antiserum at a dilution of 1∶100 (anti-alpha 7), 1∶100 (Anti-RPN10) or 1∶100 (Anti-PA26). The samples were washed and incubated for 1 h with Alexa 488-conjugated goat anti-mouse antibody (Sigma) diluted at 1∶500, as well as with DAPI (0.3 µg/ml). Stained slides were observed with an epifluorescence microscope (Nikon Eclipse E600) using a 100x objective.

### Preparation of *T. cruzi* proteasomes

We used about 6×10^8^ cells from cultures at six different periods during the differentiation process: three- and five-day-old cultured epimastigotes, epimastigotes under nutritional stress, adhered epimastigotes at 12 h and 24 h of differentiation and metacyclic trypomastigotes. The parasites were centrifuged at 7,000 g for 10 min, the pellets were resuspended in 1 ml of 20S buffer (25 mM Tris-HCl pH 7.5, 1 mM DTT, 10% glycerol, 1 mM EDTA, 1 mM leupeptin, 10 mM NEM, 1 mM PMSF, 1 mM TPCK, 1 mM TLCK, 10 µM E64) and homogenized four times in a Marconi 102/plus mixer at 27,000 rpm. Serine proteases were inhibited with PMSF, TPCK and TLCK, cysteine proteases were inhibited with E64, leupeptin and NEM. Calcium-dependent proteases were inhibited with EDTA. The samples were then centrifuged at 10,000 g for 30 min, and then at 100,000 g for 60 min, and the supernatants were recovered (soluble protein extract or crude extract)[48]. The protein content of the supernatant was determined by the BCA method [49], using bovine serum albumin (BSA) as the standard.

### Measurement of the peptidase activity of proteasomes

Peptidase activities were determined with 100 µg of crude extract from each of the six cell differentiation stages. The assay was performed by mixing each extract with the fluorogenic substrates Suc-LLVY-AMC, Cbz-GGR-AMC and Ac-YVA- AMC (100 µM diluted in 50 mM Tris–HCl, pH 7.5), at a concentration of 13 µM, in a total volume of 240 µl of reaction mixture buffer (50 mM Tris–HCl, pH 7.5, 5 mM MgCl_2_, 1 mM DTT, and 1 mM ATP). The samples were incubated at 37°C for 30 min in the presence or absence of 50 µM MG132, a proteasome inhibitor. The reaction was stopped by adding 2 ml of ethanol [48]. Chymotrypsin-like, trypsin-like and peptidylglutamyl or caspase-like peptide-hydrolyzing activities were determined by fluorimetric quantification of the fluorogenic substrates Suc-LLVY-AMC, Cbz-GGR-AMC and Ac-YVA- AMC, respectively, at an excitation wavelength of 380 nm and an emission wavelength of 440 nm, with an RF-5301PC (Shimadzu - Tokyo, Japan) spectrofluorophotometer. The results are expressed in relative fluorescence units (RFU).

### Detection of ubiquitinated proteins

For the detection of ubiquitinated proteins, western blots were carried out as described above, with an antiserum against the ubiquitin of *T. cruzi* and 20 µg of total cellular extract from each of the six stages of cell differentiation. Bound antibodies were detected by enhanced chemiluminescence (Amersham Biosciences), according to the kit manufacturer's instructions. The relative changes in ubiquitinated conjugate levels during metacyclogenesis were determined with the Scion Image program (Scion Corporation, Fredericks, MD, USA) (http://www.scioncorp.com/frames/fr_download_now.htm). Western blot results were normalized by incubating the same membrane with an antibody against the actin of *T. cruzi* as a loading control. The density of the immunoreactive bands was divided by the density of the actin band in the corresponding gel lanes. The results are expressed in densitometric units.

### Detection of protein carbonyl groups

For detection of the carbonyl groups formed on oxidized proteins, aliquots of the culture containing 5×10^8^ parasites were removed from each of the six stages during metacyclogenesis, and collected by centrifugation at 7,000 g and 4°C for 5 min. The cell pellets were washed three times in 1 x PBS and resuspended in 100 µl of lysis buffer containing 20 mM Tris–HCl (pH 8.0), 0.3 M NaCl, 1 mM PMSF, 10 µM E64, 10% glycerol and 0.1% NP-40. Cell suspensions were mixed, subjected to eight cycles of vigorous vortexing for 30 s and chilled on ice for 1 min. They were then centrifuged at 10,000 g and 4°C for 20 min. The supernatants were subjected to western blotting for the detection of protein carbonyl groups, as previously described [50]. Samples (20 µg of protein) were treated with 2,4-dinitrophenylhydrazine, subjected to SDS–PAGE in a 10% polyacrylamide gel and transferred onto a nitrocellulose membrane [51]. The membranes were blocked by overnight incubation with 2% bovine serum albumin and 5% skim milk. The membranes were then incubated with rabbit anti-dinitrophenyl antiserum (Sigma), followed by phosphatase-conjugated anti-rabbit IgG antibody (Sigma). Carbonyl-containing proteins were visualized with an NBT/BCIP reagent kit (Promega).

The relative changes in carbonyl group numbers during metacyclogenesis were determined and normalized with the Scion Image program, as described above, for the immunoblotting of ubiquitinated proteins.

### Detection of protein carbonyl groups after proteasome inhibition

Cultures of 10^6^ cell/ml epimastigotes were incubated with or without (control) 5 µM (IC_50_) [21] lactacystin. Aliquots were removed after 48 h and 72 h and the cells were collected by centrifugation at 7,000 g and 4°C for 5 min. The cell pellets were washed three times in 1 x PBS and resuspended in 100 µl of lysis buffer containing 20 mM Tris–HCl (pH 8.0), 0.3 M NaCl, 10% glycerol, 0.1% NP-40 and 10 µM lactacystin. A control was included in which the sample was not treated with 2,4-dinitrophenylhydrazine, but was subjected to SDS–PAGE in a 10% polyacrylamide gel and transferred onto a nitrocellulose membrane. The membrane was then incubated with a rabbit antiserum anti-dinitrophenyl (Sigma). Carbonyl groups on oxidized proteins were detected as described above. Normalization was performed as described above, for the immunoblotting of ubiquitinated proteins.

### Statistical methods

A multifactor ANOVA, followed by Fisher's test, was used for statistical analysis. The least significant difference (LSD) test was used to compare mean values. Means were considered to be significantly different if p <0.05.

## Supporting Information

Figure S1 **Peptidase activities were determined by fluorimetric quantification of the hydrolysis of various specific fluorogenic substrates: chymotrypsin-like (A), trypsin-like (B), and caspase-like (C) proteases.** Black bars represent the activity and white bars represent the inhibition of the activity in each group of parasites analyzed in three independent experiments: three-day-old cultured epimastigotes (Epi 3d), five-day-old cultured epimastigotes (Epi 5d), five-day-old cultured epimastigotes under nutritional stress (Epi ST), adhered epimastigotes after 12 h of differentiation (Adh 12 h), adhered epimastigotes after 24 h of differentiation (Adh 24 h) and metacyclic trypomastigotes (MT). Results are shown as means of three independent experiments ± SD.
(TIFF)
