# Supplementary figures and images for: Analysis of Proteasomal Proteolysis during the In Vitro Metacyclogenesis of Trypanosoma cruzi
Source: PLoS One. 2011 Jun 17;6(6):e21027. doi: 10.1371/journal.pone.0021027 (PMC3117861; doi:10.1371/journal.pone.0021027)

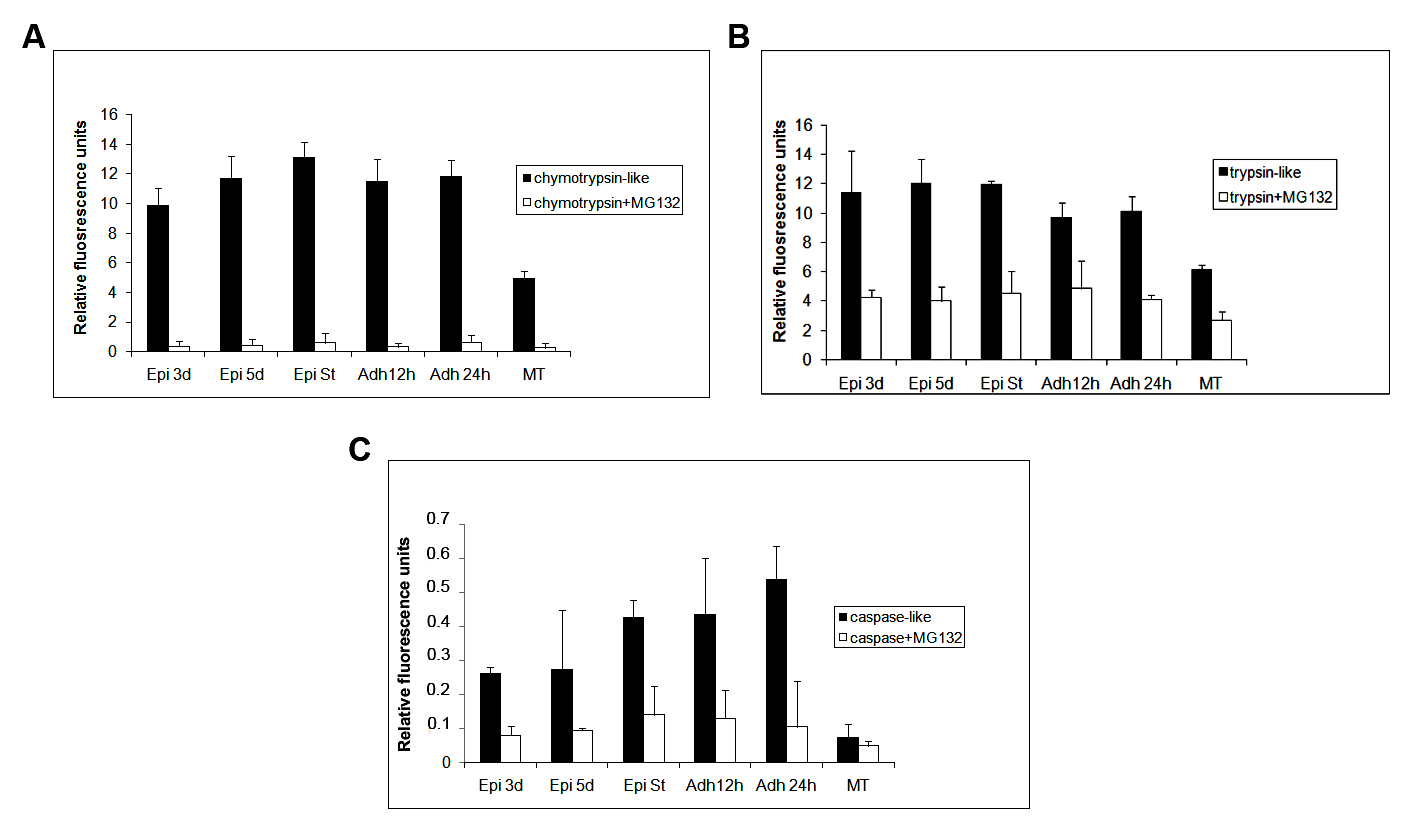

Supplement: Figure S1 — Peptidase activities were determined by fluorimetric quantification of the hydrolysis of various specific fluorogenic substrates: chymotrypsin-like (A), trypsin-like (B), and caspase-like (C) proteases. Black bars represent the activity and white bars represent the inhibition of the activity in each group of parasites analyzed in three independent experiments: three-day-old cultured epimastigotes (Epi 3d), five-day-old cultured epimastigotes (Epi 5d), five-day-old cultured epimastigotes under nutritional stress (Epi ST), adhered epimastigotes after 12 h of differentiation (Adh 12 h), adhered epimastigotes after 24 h of differentiation (Adh 24 h) and metacyclic trypomastigotes (MT). Results are shown as means of three independent experiments ± SD. (TIFF) [file pone.0021027.s001.tif]
